# Prioritizing family-centered developmental care: insights from parents of children with critical congenital heart disease: a qualitative study

**DOI:** 10.1007/s00431-024-05600-9

**Published:** 2024-06-18

**Authors:** Maaike C. A. Sprong, Iza R. Zwagerman, Lotte Soeters, Martijn G. Slieker, Tim Takken, Agnes van den Hoogen, Marco van Brussel

**Affiliations:** 1 Child Development & Exercise Center, Wilhelmina Children’s Hospital, University Medical Center Utrecht, Utrecht University, KB 02.056.0, PO Box 85090, Utrecht, 3508 AB Utrecht The Netherlands; 2 Department of Pediatric Psychology, Wilhelmina Children’s Hospital, University Medical Center Utrecht, Utrecht University, Utrecht, 3508 AB Utrecht The Netherlands; 3 Department of Pediatric Cardiology, Wilhelmina Children’s Hospital, University Medical Center Utrecht, Utrecht University, Utrecht, 3508 AB Utrecht The Netherlands; 4 Department of Neonatology, Wilhelmina Children’s Hospital, University Medical Center Utrecht, Utrecht University, Utrecht, 3508 AB Utrecht The Netherlands

**Keywords:** Children, Critical congenital heart disease, Neurodevelopmental care, Parental experiences, Family-centered care, Qualitative research

## Abstract

As survivors of early cardiac surgery are at high risk of neurodevelopmental impairments, systematic health observations of children with critical congenital heart disease (CCHD) throughout childhood are recommended to enable early diagnosis and offer interventions to optimize neurodevelopment. A qualitative study using thematic analysis was performed to explore parents’ concerns, experiences, and needs regarding the development and received developmental care of their child (0–10 years) during hospital admission and beyond. Data were collected using semi-structured online interviews with 20 parents of children with CCHD. Four major themes were identified: (1) “impact of diagnosis and disease on the family-system,” (2) “parental concerns from diagnoses and beyond,” (3) “the need for information,” and (4) “the need for individualized and family-centered care.” The main themes can be divided into 13 sub-themes as impact, concerns, and needs are influenced by various impactful moments from diagnosis and afterwards.

*     Conclusion*: This study confirms the importance of early identification of neurodevelopmental problems by experienced healthcare professionals, especially in the early years when parental expectations and concerns about their child’s neurodevelopment are lower. A tailor-made family-centered follow-up program should be offered, which pays attention to both the neurodevelopment of patients with CCHD as well as the mental wellbeing of the entire family system. Furthermore, an online portal is recommended with a variety of reliable, controlled, understandable information from which parents can obtain the desired information to understand better the consequences of specific heart condition and to provide their child with the best possible guidance.

**Table:** 

**What is Known:** *• Survivors of early cardiac surgery are at high risk of neurodevelopmental impairments; systematic health observations of children with CCHD throughout childhood are strongly recommended.*	
**What is New:** *• Parents need a tailor-made family-centered follow-up program, which pays attention to both the neurodevelopment of patients with CCHD as well as the mental wellbeing of the entire family system.* *• An online portal offering diverse, trustworthy information and sources would effectively meet parents’ needs by providing accessible insights into the potential consequences of specific heart conditions and guiding them in supporting their child optimally.*

**Supplementary Information:**

The online version contains supplementary material available at 10.1007/s00431-024-05600-9.

## Introduction

Congenital heart disease (CHD) affects approximately nine per 1000 live births, accounting for 1.35 million newborns worldwide annually [1]. Around one-fourth of all CHDs are considered critical. A critical congenital heart defect (CCHD) is a type of congenital heart disease (CHD) that requires early diagnosis and generally surgical correction in the first months of life. Examples of CCHDs include hypoplastic left heart syndrome (HLHS), transposition of the great arteries (TGA), and tetralogy of Fallot (TOF) [2]. Over recent decades, cardiovascular medicine and surgery advancements have significantly reduced mortality rates, allowing most patients to survive into adulthood [3, 4]. Survivors of early cardiac surgery are at high risk of neurodevelopmental impairments, varying from mild and barely detectable to severe developmental delays, which affect cognition, behavior, motor, and language development. Motor impairments often become apparent within the first year of life, followed by developmental limitations in the other developmental areas [5, 6]. This is of concern as neurodevelopmental impairment can have a significant impact on future limited physical and educational achievements, reduced social interaction, and diminished quality of life [7–9].

Neurodevelopmental delays can be partly explained by delayed brain development and white matter injury [10, 11] which occur in 30–50% of all newborns with a CCHD [12–16]. A CCHD causes abnormal circulation patterns in the uterus that affect the delivery of oxygen-rich blood to the fetus’ brain. The brains of newborns with CCHD may, therefore, be less developed and particularly vulnerable from the start [17, 18]. Children with a CCHD must endure many medical and surgical interventions during the newborn period and into childhood, which increases the vulnerability to brain damage and adverse neurodevelopmental outcomes [17–19]. Supplementary important risk factors for adverse neurodevelopmental outcomes are genetic disorders, a complicated postoperative course, and a poor socio-economic environment [6, 19]. The exact causal pathways of abnormal neurodevelopment appear multifactorial and are not yet fully understood. 

To enable early diagnosis and offering supportive strategies to minimize or prevent neurodevelopmental impairments, systematic health observations of children with CCHD throughout childhood are strongly recommended [20, 21]. Nevertheless, a dedicated neurological follow-up clinic, as part of standard care for children who have undergone neonatal cardiac surgery, is to our knowledge still rare in European centers. In the Wilhelmina Children’s Hospital, a follow-up program: the Hart op Weg follow-up is offered to all children with a CCHD. Due to the burden on the patient of frequent hospital visits [22], as well as the medical costs associated with such a structured follow-up, the availability of a tailor-made follow-up protocol is essential for those who need it most. To meet the needs of parents, the follow-up should be evaluated through qualitative analyses of the concerns, needs, and experiences of parents. Therefore, the primary objective of this qualitative study is to investigate the concerns, needs, and experiences of parents of children with a CCHD concerning the neurodevelopment and received developmental care of their child. Hereby, the parents’ needs were taken into account and understood their experiences with the current follow-up. The research question is twofold: (1) What are the concerns and needs of parents of children with a CCHD regarding their child’s neurodevelopment? (2) To what extent did developmental care during hospitalization and after discharge meet the needs of parents? This information would offer valuable insights for providing and improving developmental care practices tailored to the needs of children and families.

## Methods

### Design and setting

A qualitative study using semi-structured online interviews was conducted among parents of children with a CCHD, surgically corrected, at the Wilhelmina Children’s Hospital in Utrecht, The Netherlands. The guideline of “*consolidated criteria for reporting qualitative research*” (COREQ) was used to report this study [23]. According to the Ethics Committee of the UMC Utrecht, this study was not subject to the Medical Research Involving Human Subjects Act (WMO; METC NedMec reference number 22/744). All parents gave written consent and acknowledged that they would not be identified in this paper because all names were removed to protect anonymity.

### Sample and recruitment

Parents of children with CCHD (TGA, SVP, TOF, or other CCHD were eligible to participate if (1) their child was born after 1 July 2011, (2) had surgery in the first 6 months of their life using cardiopulmonary bypass, and (3) participate or participated in the “Hart op Weg” follow-up program. Parents were excluded from this study if they did not speak or understood the Dutch language or had no internet access. The coordinating investigator (M.C.A.S.) purposely selected the sample to identify and select parents who could provide the best information to achieve the study’s objectives. The following criteria were selected to obtain data: the sample consists of parents of children in different age groups: 0–3 years old, 4–7 years old, and 8–11 years old. In each age group, at least one parent of a child with TGA, SVP, TOF, and other CCHD was interviewed. To avoid selection bias, participants were selected by lot and contacted by phone by the coordinating investigator. When interested in participating in the study, parents received the patient information letter and an informed consent form and were asked to return the signed informed consent form if they agreed to participate. After receiving the signed informed consent form, parents were contacted again to make an appointment for the interview.

### Follow-up Hart op Weg outpatient clinic

**Fig. 1:**
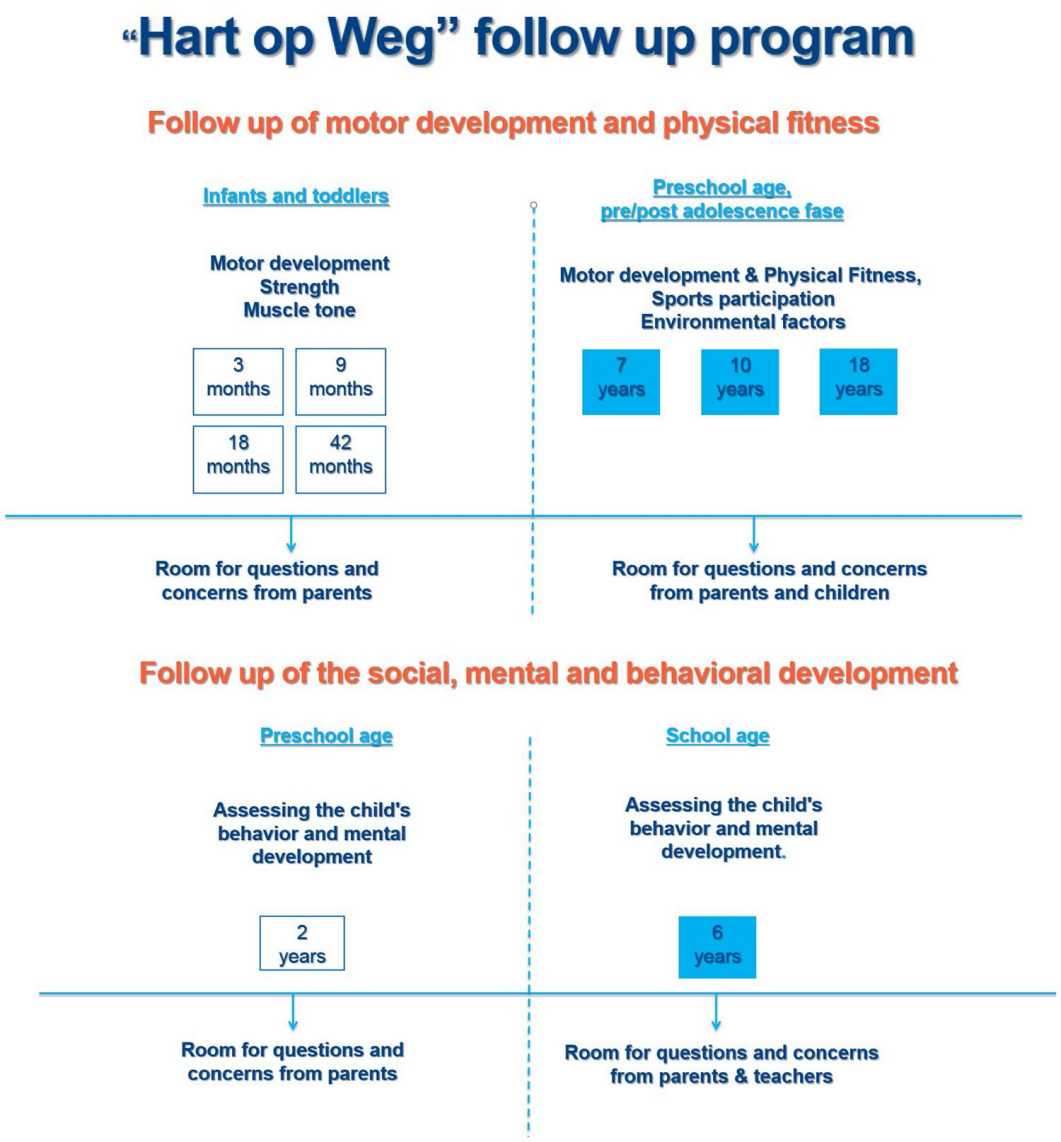
Overview of the “Hart op Weg” outpatient clinic

Since 2011, the Hart op Weg follow-up program is offered to all children with a CCHD. This follow-up program consists of a structured neurodevelopment evaluation at predetermined moments in the development of children with a CCHD (see Fig. 1) and aims to identify developmental problems as early as possible and to initiate interventions or additional comorbidity diagnostics if needed. Quantitative research (METC.NR. 13/442) into the prevalence of development problems and risk factors for abnormal development in children with CCHD was performed [16, 24–26].

### Data collection

Data were collected between January 2023 and July 2023 using semi-structured interviews with parents focusing on their concerns, expectations, needs, and experiences to explore to what extent current care meets the needs of parents. The interview was compiled based on literature [27, 28] and expert opinion. Before the pilot interview, the interview guide was presented to an independent parent of a child with a CCHD and asked whether the questions were clear and complete. The audio- and video-recorded interviews were conducted using communication platform M.S. Teams to avoid extra burden of hospital visits. The duration of the interviews was approximately 60 min. The researcher who performed the interviews (I.R.Z.) was a psychologist trained in qualitative interview techniques, and a pilot interview was performed. Data were collected until data saturation was reached [29]. Details regarding the interview questions are depicted in Appendix 1. Family demographics of the study participants, such as gender, age, education level, the child’s cardiac diagnosis, and additional support, were collected during the interviews (see Table 1).

**Table 1:** Characteristics of interviewed parents and their children

**Interviews mother (M) and father (F)**	**Age**	**Parent’s educational level**	**Child’s gender**	**Child’s cardiac diagnosis**	**Antenatal diagnosis** **Yes/no**	**Number of cardiac surgeries**	**Child’s age group**	**Child receiving extra developmental support**
M0	39	High	Female	Others	no	5	4–7	-
M1	42	High	Female	TOF	yes	1	4–7	-
**F2**	39	High	Male	TGA	yes	1	8–11	PPT is involved for fine motor difficulties
M3	38	Middle	Male	SVP (HLHS)	yes	3	0–3	A dietitian is involved for failure to thrive
M4	36	High	Female	TGA	no	1	4–7	-
M5	36	High	Male	SVP (HRHS)	yes	3	8–11	Diagnosed with dyslexia Receives dyslexia counseling and PPT for sports injury
M6	26	Middle	Male	SVP	yes	3	4–7	School guidance service involved for behavioral problems at school
M7	34	Middle	Male	TGA	yes	1	0–3	-
F8	37	High	Male	TOF	yes	3	0–3	-
M9	36	Middle	Female	Others	no	1	0–3	-
M10	41	High	Male	TOF	yes	2	8–11	Diagnosed with ADHD PPT for fine motor problems
M11	32	Middle	Male	SVP (HLHS)	yes	3	0–3	CP, but currently no extra support Regular follow-up in the rehabilitation center Epilepsy
M12	37	High	Female	Others	yes	1	4–7	-
M13	43	High	Female	SVP	yes	3	8–11	Special education for learning difficulties
M14	31	Middle	Male	SVP (HLHS)	yes	3	4–7	PPT for fine and gross motor difficulties Epilepsy
M15	45	Middle	Female	TOF	yes	1	8–11	-
M16	30	Middle	Female	TGA	yes	1	0–3	-
M17	42	High	Male	TOF	yes	2	4–7	Diagnosed with autism and follows special education; Cesar’s therapist is involved for fine motor difficulties, systemic therapy for parents, and foster care is involved once a month to relieve parents
F18	40	Middle	Male	TGA	yes	1	4–7	A psychologist is involved for behavioral problems
M19	50	High	Male	TGA	yes	1	8–11	-

Educational level was classified as low, middle, or high (The Hague, the Netherlands: https://www.cbs.nl/)

*SVP* single-ventricle physiology, *PPT* pediatric physical therapist, *CP* cerebral palsy, *ADHD* attention deficit hyperactivity disorder

### Data analysis

Data were analyzed using thematic analysis, according to Braun and Clarke [30]. Data were coded inductively and inclusively to ensure that context was preserved. The qualitative data analysis software program NVivo 11 (QSR International: Melbourne, Australia) was used for data management during the analysis. The following phases were performed: phase 1 familiarizing the data. Before coding, all audio- and videotaped interviews were transcribed verbatim by M.S. A summary of the manuscripts was made, and manuscripts were read and reread. Member checking was performed by offering participants the opportunity to add comments and corrections to the transcripts. Phase 2, generating initial codes: individual narratives of the parents were coded from in vivo codes to subcategories and put into categories to generate themes. To ensure rigor and trustworthiness, this was performed by two independent researchers (I.R.Z. and M.C.A.S). In the third phase, the codes were systematically coded into themes and subthemes (I.R.Z. and M.C.A.S.). In phase 4, the themes were reviewed with a third and fourth reviewer (M.v.B., A.v.d.H., I.R.Z., and M.C.A.S.). Finally, in the fifth phase, themes were defined and named. To improve the clarity and relevance of the themes, the sets of categories were adjusted and rearranged to refine the themes further. Disagreements among researchers were discussed until a consensus was reached.

## Results

Twenty semi-structured interviews were completed with three fathers and 17 mothers. All parents agreed with the summary, and no additions were required to be included in our analyses. Therefore, no changes were required. Characteristics of the participants and their children are provided in Table 1. Themes and categories were developed. The main themes that parents of children with CCHD reported were: (1) “impact of diagnosis and disease on the family system,” (2) “parental concerns from diagnoses and beyond,” (3) “the need for information,” and (4) “the need for individualized and family-centered care.” The main themes can be divided into several sub-themes. The results are described in detail below.

### Impact of diagnosis and disease on the family system

A CCHD appears to have an impact on various levels within a family system: (1) “impact on the patient,” (2) “impact on families (parents and siblings),” and (3) “impact on parental perceptions of child vulnerability and parenting style.”

#### Impact on the patient

Most parents indicate that their child functions above expectations and that the impact of the CCHD on the child is smaller than expected.

*It has exceeded our expectations as I never thought he could play football for so long and that he can keep it up like this. (M5)*

The impact is more significant when there are developmental problems. Accordingly, parents indicate that they are surprised that having a CCHD can influence so many areas. At the same time, they wonder whether the problems are the impact of the heart defect or whether this is innate and would also exist without a heart defect. According to their parent, children with a CCHD appear to experience merely few obstacles themselves, partly because the child does not know any better.

#### The impact on families

The impact of the heart defect may be more prominent for the parents and siblings than for the patient himself, as all parents indicated that the impact on themselves is enormous. The child’s condition can impact work, relationships, and the psychosocial well-being of parents. The impact on families is most significant in the first months to years after diagnosis, depending on the number of operations and additional problems and complications (e.g., tube feeding, protein losing enteropathy, allergies, or stagnant growth and development).

Parents describe the diagnosis, the subsequent choice to terminate or continue the pregnancy, the stress of childbirth, concerns surrounding the operation(s) and recovery, and the fear of possible additional problems as an uncertain rollercoaster phase. Parents report that they have endured this stressful roller coaster, which has lasted for months to years, in a kind of survival mode.

*Especially at the beginning, it greatly impacts your daily life. It affected his brother, who had to sleep at Grandpa’s and Grandma’s. It affects your work. I was less concentrated, especially when things were not going well. You get some tough news yourself, which also takes a toll on you psychologically, so you must learn to deal with that. Furthermore, as a couple, you also must work together again. My husband deals with it differently than I do, So, in the end, it turns our lives upside down. (M11)*

When it comes to the impact of the heart defect at the time of the interviews, several parents indicate that they always have to take the heart defect into account in their daily activities: they always have to think a few steps ahead (e.g., is there is a hospital nearby?), and they must be alert to overload or deterioration of cardiac function, and flexible in unexpected situations.

Additionally, the heart defect and associated hospital admissions and operations also have an impact on siblings. There are emotions like anger, anxiety, and sadness about the absence of parents during admissions, and older brothers and sisters are often extra alert but also concerned about the patient’s well-being. In the beginning, especially with more care-intensive children, much attention is paid to the patient, which sometimes leaves a mark on the family in the long term. Parents try to ensure that “the patient” is not given a special place within the family and that brothers and sisters receive sufficient attention. However, a CCHD must always be considered when planning activities and days out.

*There is always a piece of extra alertness and, yes, also from the oldest children. There is always that extra bit of care: “Oh X, do not get on that climbing frame or you might fall out,” and taking her into account: Where are we going? Is it suitable for X? (M0)*

#### Impact on parental perception of child vulnerability and parenting style

Parents reported that they perceive their child as more vulnerable, even though there is not always a reason to do so. In the case of more care-intensive children, it is difficult to let go of children and leave the daily care to others. These parents also indicate that this may limit their children’s actions, partly due to increased bleeding tendency due to blood-thinning medication in the event of falls and fear of overexertion. Regardless of the child’s overall functioning, parents indicate that they are overprotective, worry more quickly, and are more alert than sibling, for example, when children have a fever or a cold that they consult a doctor more quickly.

*X was slow in developing large motor skills, and I linked everything to the heart defect, everything! With every cough, every vomit, I thought, oh no, her heart! While it was just the stomach flu. That was, of course, also my perception as an overprotective parent… (M1)*

### Parental concerns from diagnoses and beyond

Results regarding parental concerns were divided into three subthemes: (1) “navigating concerns per phase”, (2) “embracing limitations in an uncertain future,” and (3) “continued concerns in the face of brain injury, declining cardiac function and future surgeries, and reduction of concerns through reassuring information.” Details are described below.

#### Navigating concerns per phase

Parents’ concerns about their child with a heart defect include short-term concerns and long-term concerns and depend on the different phases they and their child go through. At the time of the prenatal diagnosis, concerns initially lie in the long term. Concerns about the short term dominate around postnatal diagnosis, birth, the operation, and in the first years afterward. When CCHD was diagnosed prenatally, parents reported that their main concern was whether the diagnosis was operable and whether there was a good chance of everyday life and adequate quality of life afterward.

*After the diagnosis, we only asked ourselves one thing, and that was: how likely is it that he can just live a normal life? That was also the question that finally made us proceed with the pregnancy. We want him to function as much as possible as a normal child. (M6)*

The concern that the heart defect was part of an underlying genetic disorder that would affect the child’s long-term functioning was also raised after diagnosis. After the decision has been made to continue the pregnancy, the fear of loss during pregnancy, delivery, or surgery arises. Another concern was the risk of complications that could affect long-term functioning, such as oxygen deficiency and its impact on the brain. After birth, in the run-up to the operation, and the period until discharge, parents indicate that they have lived from day to day, regardless of whether the diagnosis was made antenatal or postnatal. Worries were mainly about the short term: the fear of loss and the worry about complications surrounding the operation. There were not many expectations or concerns about the long term: they were trying to keep their child alive and survive themselves. After discharge, during the baby-toddler phase, parents continue to look in the short term and do not look too much ahead "from school age towards puberty, more concerns about the long-term arise again".

*Initially, you are not concerned with the future at all. You are only concerned with the fact that my child has a heart defect and has to undergo an operation, and you have no idea how that works or what it is like. You have no room to think. It is still a life-or-death story in your head and has nothing to do with the future. You are just surviving and making sure your child stays alive. That is your biggest priority, and then you do not think about the future yet. You do not even want to think about the future because you are happy that you can take her home from the hospital. (M0)*

#### Embracing limitations in an uncertain future

After discharge relief and joy prevail that their child is still alive, or their worries have not come true. Furthermore, parents indicate that they do not have high expectations of their child or consider functioning relative to healthy peers important, as the child’s well-being is most important in this phase, and it is unfair to compare their child with other children. Every child has a different medical history and usually develops in their own way and at their own pace.

*If she wanted to become a pilot, that would not be possible because she has that abnormality. Well, if that is it… Not a top athlete either well… I do not think she has that ambition, either. So, you know, if that is it. (M4)*

At school age, when there is more comparison with peers, when difference with peers increases, and towards puberty, more concerns and questions arise about the future. Concerns are reported regarding future functioning: “what can my child physically handle in the future,” “what can we expect from him,” and “ what problems will my child encounter when he gets older.” Also, concerns terms of mental well-being: “how much impact will the heart defect have on my child when he or she becomes a parent?” and concerns regarding independency are mentioned. Most concerns do not dominate but come up sometimes for most parents.

#### Continued concerns in the face of brain injury, declining cardiac function and future surgeries and reduction of concerns through reassuring information

Parents of children who have ended up in a resuscitation setting or who have had brain damage detected on postoperative MRI are more concerned about the future in terms of development and overall functioning in the period shortly after the operation.

*In the beginning, mainly whether he would have a syndrome and what it would look like after birth. A bit of uncertainty, but later, after the operation and the cardiac arrest, more of: what will the future look like for him in terms of development? (F8)*

Concerns about future functioning are also more significant and longer present among parents of children, of which it is expected they need cardiac interventions in the future. They wonder what the impact of declining heart function or new risky interventions on the child’s functioning will be.

*With every operation, you will be told in advance what can go wrong, the risks, etcetera. However, yes, there were a lot of them… I have to say that the biggest concern for me was that she could not do anything afterward, that she had suffered from a lack of oxygen or that she could not walk or talk anymore, that you, yes, no, having a child back… that was my biggest fear, with every operation. (M13)*

On the other hand, concerns are reduced in all phases by the absence of a genetic abnormality, a successful outcome of the operation, a good MRI, good functioning compared to peers, and a good assessment by a doctor or during follow-up assessments.

### The need for information

The results reflect three aspects of parent’s need for information: (1) “the varying need for information and information source,” (2) “informed future: setting expectations with phased and individualized insights,” and (3) “insight into the neurodevelopment of children with heart defects and their incomparability with (healthy) peers.”

#### The varying needs for information and information sources

The interviews indicated that the need for information differs significantly between parents and, like concerns, changes by phase. When the heart defect is diagnosed antenatally, there is first a need for information on whether the heart defect is operable. Subsequently, information about the prospects of children with similar heart defects, both positive and negative, is needed to make a sound decision regarding continuing the pregnancy and to provide hope and reassurance.

*I have looked up many things, which can sometimes confuse. You wonder: Is this article and what I am reading now correct? Anyway, you also take some advice from other parents about the experiences you read. (M9)*

When it was decided to continue the pregnancy, the baby was born, and the operation completed, the need for information about the prospects of their child declines, and they do not look too much ahead. Information about future functioning can create unjustified expectations, but also unnecessary worries. Parents indicate that they have questions but realize that no one can predict how their child will develop. More questions arise about the heart defect’s future and overall functioning from school age onwards.

*Now I would like to know: what are the life expectancies and experiences? Because we are now several years further, I am always curious about how children like that develop. How long do they live? And, of course, with age, more questions arise… Sometimes, I think about puberty. Can she get her period? Can they have children? Do you know all kinds of things for the future? That remains an unknown terrain for me, and maybe I will… Yes, I think she will also come up with all those questions herself later. But I know little about the future; what are the statistics, so to speak. (M13)*

#### Informed future: setting expectations with phased and individualized insights

Information needed in the hospital admission run-up is about what lies ahead in the short term. Parents indicate their need for preparation for the admission and operating room using photo albums or guided tours. “What can you expect during such a hospital admission? What does the Intensive Care Unit and nursing department look like, what facilities are there, and what can you find where?”.

*She rang the doorbell, and she said: I have something very confrontational for you, but it will help you, she then arrived with a booklet with photos of her child's open-heart surgery, and I thought: What is happening here? But when X was done and went from the OR to the ICU, I knew what to expect… I liked that. I could look at my child, and I did not have to look at all those tubes and wires because I knew what that image looked like, things like that, you want that. That is very confrontational, but it will help you so much. (M0)*

In addition, several parents indicate that they need more information about possible secondary problems to be better prepared for what might come. Parents mention here the possible dependence on tube feeding after surgery, the impact on relationships and work, and the possible impact of hospitalization and surgery on both the parent and the child, such as stress, fears, nightmares, and trauma and how to deal with this as a parent.

Parents also indicate that they still need periodic information in the form of a mailing, a blog, or a newsletter with the latest research results about the type of heart disease their child suffers from and information about what to expect regarding neurodevelopment and neurodevelopmental problems and how they can support their children as best as possible. Finally, parents indicate their need for reports of their child’s assessment results in understandable language to be able to read back and share with, for example, school and other stakeholders.

*You would prefer to receive clear feedback on paper about what has been done and the results. And in such a way that you can pass it on directly to the childcare or the school so that they know better about “Hey, what is going on? what have they been looking at?” Should we do anything about this as a school or day-care? Or can we help the child with something?” I think that as parents, you can easily reread it, without medical terms. (M6)*

#### Insight into the development of children with heart defects and their incomparability with (healthy) peers

There is a general feeling that children with a heart defect cannot be compared to healthy children or siblings and that his or her medical background should be considered when assessing development. If developmental problems arise, there is a solid need to be able to place these in the light of his or her heart defect. Many parents, therefore, wonder whether the problems they encounter are more common in children with a heart defect.

*And that might take away some concerns, if you know this could be part of her heart defect, then you think: okay, then that is it! Yes, that also gives you a bit of peace and security. (M9)*

On the other hand, there is a realization that as a parent, you will never know whether the problems would not have existed without the heart defect. However, the knowledge that the developmental problems their child experiences are common provides recognition, relief, resignation, understanding, trust, and confirmation.

*Concentration, a full head, yes, stimuli are difficult: he is quite bothered by noise stimuli. Yes, we cannot say in black and white: that is due to the heart defect, so you treat him in the sense of ADHD, and it is nice that you can act that way towards him. You can never say whether it is a direct consequence, right. (M10)*

### The need for individualized and family-centered care

Regarding parental experiences and needs for neurodevelopmental care during hospital admission and after discharge, four subthemes emerged: (1) “setting and shifting priorities”; (2) “the essential role of a customized, multidisciplinary follow-up program across childhood”; (3) “promoting empowerment and trust”; and (4) “family well-being and the need for aftercare for parents and siblings.”

#### Setting and shifting priorities

In the phase surrounding neonatal cardiac surgery, there is a particular need to strengthen the parent–child relationship. Attention to attachment, facilitating breastfeeding, involvement, and thinking along with parents in care are considered very valuable. The focus should be on comfort, survival, and recovery. Neurodevelopment is not a priority during this period.

*When I am in the hospital, for my child who has had a heart operation, I would prefer that they take care of that. So I also think that care should be focused on correcting her heart, and I think it is very good that attention is also paid to other matters, but that is not why she is there. (M4)*

However, parents indicate that good medical clinical care has contributed to the functioning of their child in the long term. Neurodevelopmental care and the availability of developmental experts, e.g., speech therapy, physiotherapy, and pedagogical care, are considered more important for more prolonged admissions or admissions are experienced as necessary, as well as the distraction and facilitation of being a child by giving information, tips, and tricks to parents about what they can offer their child to promote development as much as possible during hospital admission.

*Where should I start? What should I do? Okay, she will sport, but is that enough? And what kind of sport is best for her? More focused... more advice focused on what suits her. (M13)*

#### The essential role of a customized, multidisciplinary follow-up program across childhood

*After discharge, there is a need for a more holistic approach to their child at the cardiology clinic, they examine his heart, conduct an ultrasound, and measure and weigh it. That heart is just a machine being fixed there, but it is a heart. Around it, there is a child doing all kinds of things. This is what I think: Yes, that is what the “Hart-op-Weg” outpatient clinic is focusing on – the bigger picture and further development. Every child must be monitored, not just regarding their heart, but also psychologically, developmentally, and the rest of the system, so to speak… from the machine. (M17)*

There is a common need for a multidisciplinary, individual, customized follow-up program, where healthcare professionals monitor how their child functions and determine whether there is a need for additional diagnostics or support should be involved. When the child is doing well, there should be room for a shorter route or temporary follow-up stop with an option to resume or easily contact us if they have any questions or concerns. Parents prefer to assess their child by experienced developmental experts who know the child and the nature of their heart defect above a general practitioner. Parents value that their child is assessed in the light of his heart defect and that specific advice is given, taking their heart defect into account.

*Well, very much of added value, because in our environment, such as a GP or regional hospital, they simply not know that much about heart defects, so they don’t watch and give advice in that way. And because many heart patients come together in the WKZ you can compare much better. You do not have a standard of healthy children but the standard of children with a CHD and… well, it really matters a lot how that’s compared. (M5)*

Furthermore, parents need guidance in providing extra support and customized tips and tricks for daily life to support their child’s optimal development and how they can support children as best as possible.

*Where should I start? What should I do? Okay, she will sport, but is that enough? And what kind of sport is best for her? More focused, more advice focused on what she, what suits her. (M13)*

According to parents, development should be assessed at fixed moments around developmental milestones and other important milestones, such as attending school and choosing a sport. Lastly, accessible availability of health care professionals between appointments is appreciated.

#### Promoting empowerment and trust

Regardless of concerns about the child’s functioning, individual attention to the child’s functioning contributes to the need for confirmation and support. The follow-up meets the need for a place where someone thinks along, and questions and concerns can be discussed. The fact that healthcare professionals are available who watch and think along so that timely action can be taken gives parents relief. The regular checks provide reassurance and remove concerns, and positive reviews give hope for the future. If parents’ positive image is confirmed, it also gives them more self-confidence.

*Yes, sometimes you just want confirmation of something. Or what a professional says about it because even though you hardly ever deal with it, there is always a bit of concern in your mind: if he is doing well and things like that. (F18)*

According to parents, assessing the capabilities of older children also gives the child self-confidence and contributes to the feeling of being no different from other children.

*A child can show during these tests that he is doing well, which is a huge incentive for the child. (M19)*

#### Family well-being and the need for aftercare for parents and siblings

There is a strong need for attention to parents’ psychosocial well-being both during admission and afterward, with attention to possible traumas and guidance to give everything a place. Parents indicate that their well-being is essential to provide their children with the best possible guidance. Several parents suggest a structural follow-up meeting a few months after discharge, when peace has returned, to identify what is needed regarding psychosocial guidance for parents.

*Maybe one more time for the parents, a conversation with a psychologist or something like that. Yes, yes, initially once, and then look from there: what extra care might be needed? (F18)*

Parents also indicate that they need attention for the well-being of siblings. Furthermore, there is a need for information, tips, and tricks on how parents can guide, involve, and inform their other children about the situation and how they can respond to questions and concerns from siblings.

## Discussion

Four main themes were identified regarding the parents’ concerns, needs, expectations, and experiences concerning the development and developmental care of their child with a CCHD: “Impact of diagnosis and disease on the family system,” “Parental concerns from diagnoses and beyond,” “The need for information,” and “The need for individualized and family-centered care”. Both impact, concerns, and needs vary in early years, differ per phase, and are also influenced by various impactful moments from the moment the diagnosis is made and afterward, such as birth, surgery, recovery, and the period thereafter in which more becomes clear about the long-term functioning of the child compared to peers. This is in line with previous findings [31, 32].

### Impact of a CCHD on the patient and the need for a tailor-made follow-up program by experienced healthcare professionals to enable early interventions and to improve long-term outcomes

A striking finding of this study was that, although many patients receive additional developmental support (see Table 1), parents indicate that the impact of CHD on their child was limited. Parents indicate, especially in the first years, that their child functions beyond expectations. An explanation for this may be that within this study, the majority (17/20) already knew antenatally that their child would be born with a CCHD. Although parents in this and other studies describe this process as a very stressful situation, these parents had the opportunity to be informed about what they could expect from postnatal treatment before they decided to continue or terminate the pregnancy. A good quality of life was most important to them, and they took for granted possible limitations and the prospect that their child might not be as functional compared to healthy peers. This finding, combined with adjusted expectations, poses a risk of late recognition of developmental delays by the parents.

Although parents indicate that it is not important how their child functions compared to other children, they stress the importance that their child can participate in daily activities with peers. A prominent issue, in the study of Knowles et al. concerning the self-reported health experiences of children with CCHD themselves, was their sense of belonging to their peer group, which was referred to as “fitting in” or “joining in.” Children associate not being able to undertake the same physical acticities as peers with social exclusion [33]. As fine motor difficulties can lead to educational problems, and gross motor difficulties can complicate participation in exercise and sports and result in a sedentary lifestyle and social isolation from peers [34], early identification of developmental problems is vital to enable early interventions at the earliest possible stage.

Early identification of neurodevelopmental problems is essential as the brain’s ability to change in response to experiences is most significant in the first years of life. As early life events and experiences can influence the pattern of brain architecture, which is critical to establishing a solid footing for development, the most significant opportunities to influence development and to prevent social isolation later in life lie in the first few years [35, 36]. Parents unanimously confirm the importance and added value of structured monitoring of their child’s neurodevelopment [21] by experienced healthcare professionals as it meets their need for guidance, monitoring, and confirmation, recognition, and relief, regardless of whether there are concerns about development.

Lastly, Knowles et al. also showed that children with a CCHD are mainly afraid of “being different,” and striving to be the same as their healthy peers seemed central to their identity [33]. Hospital visits gave the feeling of being different. To prevent as many unnecessary hospital visits as possible, parents advocate that children are seen and assessed not too often but around essential milestones in a tailored follow-up program that examines for each patient individually how the follow-up should be carried out in terms of intensity, but also which healthcare providers should be involved.

### Impact on parental perceptions of child vulnerability and parenting style

Despite parents indicating that the impact of their child was limited, one of the subthemes from our study was the impact of the heart defect on parental perceptions of child vulnerability and the resulting overprotective parenting style. An earlier study from our study group in which higher rates of perceived child vulnerability (7.3%) and parental overprotection (21%) were established confirms this finding [37]. These parenting factors could negatively influence their child’s development as a significant correlation was found between parental perceptions of child vulnerability and physical activity and sports participation levels of their child. McCusker et al. also emphasized that parenting factors were more important than the severity of heart disease in predicting and explaining psychosocial and neurodevelopmental outcomes [38]. This underlines the importance of good information provision to parents by healthcare professionals about the essence of mastering age-specific motor skills to enable participation with peers in sports and games. Furthermore, counseling parents regarding the risks and benefits of physical activity and periodic information provision about what is possible and permitted with a CCHD should also be part of follow-up.

### Impact on family system and the need for family-centered care

Although the impact on the child with CCHD is less than expected, several parents indicate the impact on themselves. The enormous impact of having a child with a CCHD is endorsed in several other studies [31, 32, 39, 40]. Parents in our study may have taken less account of the impact on themselves, especially since parents in this study repeatedly indicated that the health and wellbeing of their child were considered most important, especially in the critical period.

As parents in our study also indicate the burden of a CCHD on their other children, they expressed a need to ensure that siblings receive the support they need. Siblings of children with CCHD experience worry and uncertainty and feel additional responsibility as also found by a recent study from Bichard et al. [41]. Having a sibling with a CHD might also affect children’s emotions, behaviors, school functioning, quality of life and health whereby the severity of CHD negatively affects siblings [42]. Given the major impact of a heart defect on parents, siblings, and family processes, rather than disease or surgical factors, in predicting outcomes [37, 38], a better understanding and addressing the needs of the entire family system might promote long-term outcomes. Healthcare professionals need to be aware of the impact of CCHD on the entire family system, and screening for psychological distress of parents and siblings of children with CCHD should be part of standard follow-up care. The parents interviewed and several other studies confirm and endorse the importance of family-oriented care [21, 31, 32, 39, 40, 43–45].

### The need individualized information provision

A final important finding was parents own need for information, which is confirmed in several previous studies [31, 45, 46]. The need for information varies between interviewees and depends on the different phases parents go through from diagnosis to the time of the interview. Most parents indicate that they regularly search on the Internet for new or additional age- and condition-specific information and experiences of other parents with the same defect. Other studies among parents of children with CHD confirm this finding [46–48] and the finding that information is not easily findable, often too complex, insufficient, or unspecific, and of moderate reliability and quality [47–50].

This study and Bratt et al. [46] support the availability of an online, evidence-based patient information portal for parents. The information portal from the pilot study of Etnel et al. [50] could be further developed and expanded to be more specifically aimed at parents of children with a CCHD. Such an information portal could be used (1) to support physicians in informing and communicating with their patients; (2) to enable parents to collect reliable and understandable information as needed, with both facts and figures about life expectancy, joint problems, and the prospects of children with specific heart defects; (3) to share information about how development usually proceeds and empower parents with tips and tricks to support and optimize their child’s neurodevelopment despite the burden of the heart defect; (4) to share more general practical information regarding relationships, work, childcare, and available support services; and (5) to facilitate contact with and support from parents with similar experiences and patient experts.

This study has several limitations. As the oldest children are now 12 years old, we cannot rule out recall bias about the period surrounding the diagnosis, operations, and early childhood. Furthermore, fathers were underrepresented, and mothers of boys were overrepresented, which could have impacted the results. The interviewed parents represent a specific demographic from a single academic pediatric hospital in Western Europe, characterized by a predominance of white, highly educated parents with significant affluence. Moreover, healthcare expenses for children are covered in the Netherlands, which may not be the case in other centers. Consequently, there may exist divergent needs and circumstances in alternative settings.

As a result, the generalizability of the findings is limited.

An essential strength of this study is that, to our knowledge, this is the first study to evaluate an existing follow-up program in children with CCHD. By prioritizing a patient-centered approach, it bridges the gap between medical perspectives and familial priorities, contributing to more collaborative and effective pediatric cardiology care. To get the broadest possible picture of the experiences, concerns, and needs, we included children with different CCHDs in several age bands, including children with genetic comorbidities. Lastly, participants were selected by lot before they were approached, and willingness to participate was high, which made selection bias less likely.

## Conclusion

This study confirms the importance of early identification of neurodevelopmental problems by experienced healthcare professionals, especially in the early years when parental expectations and concerns about their child’s neurodevelopment are lower. A tailor-made family-centered follow-up program should be offered that pays attention to both the neurodevelopment of the patient with CCHD as well as the mental wellbeing of the entire family system surrounding the patient with CCHD. To develop and improve appropriate early interventions to improve long-term outcomes after early cardiac surgery, a deeper understanding of patients’ and parents’ experiences and needs is needed. Therefore, more extensive research on family perspectives and risk and protective family factors is needed.

Furthermore, the availability of adequate individualized information provision is vital. In addition to the easily accessible availability of healthcare professionals to share concerns and questions, an online portal with a variety of reliable, controlled, understandable information and sources should be available from which parents can obtain the desired information to understand better the consequences of specific heart condition to provide their child with the best possible guidance.

### Supplementary Information

Below is the link to the electronic supplementary material.

Supplementary file1 (PDF 76 KB)

## Data Availability

The data that support the findings of this study are available from the corresponding author upon reasonable request. Data are located in controlled access data storage at Wilhelmina Children's Hospital.
