# Supplementary material for: Prioritizing family-centered developmental care: insights from parents of children with critical congenital heart disease: a qualitative study
Source: Eur J Pediatr. 2024 Jun 18;183(9):3863–76. doi: 10.1007/s00431-024-05600-9 (PMC11322194; doi:10.1007/s00431-024-05600-9)
Supplement: Supplementary file 1 — Supplementary file1 (PDF 76 KB) [file 431_2024_5600_MOESM1_ESM.pdf]

Appendix 1 Interview guide

The interviews usually began with a general question. During the interview probes, prompts, and follow-up questions were used for clarification, such as: "Can you elaborate on that?", "What made you not worry?" or "What were these worries about?" and "What would you have liked to have gotten more information about?" and when would you have liked to receive this information? The interview ended with an open topic that parents wanted to discuss before finishing the interview.

General question
- How have you experienced the care at the WKZ for your child so far?

Parents' expectations and concerns regarding development.
- How does having a child with a congenital heart defect affect the daily life of your child and your family?
- What were you most concerned about after the diagnosis?
- Can you tell us something about the expectations or concerns you had at that time regarding your child's future with regard to development (e.g. motor, mental, behavioural, physical fitness (condition) and (sports) participation)?
- Have these expectations or concerns been met?
- Do these concerns still exist today?

The experiences and needs of parents with regard to development-oriented care during admission
Your child has been admitted to the WKZ one or more times
- What are your experiences with the attention paid to your child's development during these hospital admissions
- What would you like to see improved in development-oriented care during admission? For example, are there matters that could have received more attention during these recordings?
- Are there any aspects of development-oriented care during admissions that you have experienced as positive and can you give examples of this?
- Would you have liked more information about the (expected) development (problems) of your child in the longer term?

The experiences and needs of parents with regard to development-oriented care after discharge
- Can you indicate how you experienced the visits to the neurodevelopmental follow up outpatient clinic? Can you explain this?
- To what extent could you discuss your questions and concerns about the development?
- To what extent were your questions/concerns addressed?
- Can you tell us something about your experiences with development studies and the communication about the results? Did the results of these studies influence your image of your child and why?
- Can you tell me something about the advice given during or after these consultations?
- Can you say to what extent the follow-up was of added value or helpful for you and your child? Can you explain this?

The needs of parents with regard to development-oriented care during the heart on the road follow-up
- Are there matters that could have received more attention during the inspections?
- What do you think the follow-up should look like and who should be involved?
- Where and when would this care ideally take place
- What would you like to see improved in the neurodevelopmental follow up outpatient clinic? (Do you have any tips or advice)
- What would you like to compliment the follow-up on?
- Would you like to say something that has not been discussed but that you still consider important to say?
